# Excessive Effects of Extreme Energy Levels on Lipid Metabolism in Ningxiang Pigs: Insights from Gut Microbiota and Glycerophospholipid Metabolism

**DOI:** 10.3390/nu17233648

**Published:** 2025-11-21

**Authors:** Jiayi Chen, Yongmei Wu, Jianhua He, Yaodong Wang, Min Wang, Yifei Lu, Fengming Chen, Yurong Zhao

**Affiliations:** 1 Hunan Provincial Key Laboratory of the Traditional Chinese Medicine Agricultural Biogenomics, Changsha Medical University, Changsha 410219, China; jiayi@csmu.edu.cn (J.C.); wu@csmu.edu.cn (Y.W.); luyifei0825@163.com (Y.L.); 2 College of Animal Science & Technology, Hunan Agricultural University, Changsha 410128, Chinasxllwyd@gmail.com (Y.W.); 13627411846@163.com (M.W.)

**Keywords:** excessive energy, restrictive energy, hepatic lipid metabolism, gut microbiota, Ningxiang pig

## Abstract

Objectives: This experiment investigated the response of carcass composition, digestive function, hepatic lipid metabolism, intestinal microbiota, and serum metabolomics to excessive or restrictive dietary energy in Ningxiang pigs. Methods: A total of 36 Ningxiang pigs (210 ± 2 d, 43.26 ± 3.21 kg) were randomly assigned to three treatments (6 pens of 2 piglets each) and fed a control diet (CON, digestive energy (DE) 13.02 MJ/kg,), excessive energy diet (EE, 15.22 MJ/kg), and restrictive energy diet (RE, DE 10.84 MJ/kg), respectively. Results: Results showed that EE significantly increased the apparent digestibility of crude protein and total energy (*p* < 0.01), as well as the activities of jejunum neutral protease and ileal lipase (*p* < 0.05). With the increase in energy level, the apparent digestibility of ash, dry matter, and ether extract significantly increased (*p* < 0.01). RE significantly increased high-density lipoprotein cholesterol (HDL-C) content, significantly decreased triglycerides (TG), free fatty acid (NEFA), and total cholesterol (TC) contents, and up-regulated lipoprotein lipase (*LPL*) mRNA expression in the liver (*p* < 0.05). EE significantly increased the hepatosomatic index, the contents of low-density lipoprotein cholesterol (LDL-C) and total bile acids (TBA), and significantly up-regulated the mRNA expression of lipogenic genes acetyl-CoA carboxylase (*ACC*), fatty acid synthase (*FAS*), and sterol regulatory element-binding protein-1C (*SREBP-1C*) in the liver (*p* < 0.05). The abundance of *p_Firmicutes* was significantly increased and the abundance of *p_Bacteroidetes* was significantly decreased in test groups, while the ratio of the two was significantly increased in the RE group (*p* < 0.05). EE also significantly increased the abundance of *g_Clostridium_sensu_stricto_1* (*p* < 0.05). The identical serum differential metabolites between the EE and RE group belong to phosphatidylcholine (PC), mostly being up-regulated in the EE group and down-regulated in the RE group (*p* < 0.05), one of which was mapped to the pathway of glycerophospholipid metabolism (KEGG ID: C00157). The relative content of serum trimethylamine N-oxide (TMAO, a microbial metabolite) was significantly decreased in the EE group (*p* < 0.05). Conclusions: The findings suggest RE had no obvious negative effect on carcass traits of Ningxiang pigs. Apart from exacerbated body fat deposition, EE promoted fat accumulation in the liver by up-regulating the expression of lipogenic genes. Dietary energy changes affect hepatic bile acid metabolism, which may be mediated through the glycerophospholipid metabolism pathway, as well as disturbances in the gut microbiota.

## 1. Introduction

Ningxiang pig is a high-quality local pig breed in China with high content of body fat and intramuscular fat (IMF). Although its meat has received high recognition, defects such as slow growth and low carcass leanness have limited the promotion of breeding and market acceptance of Ningxiang pigs, resulting in the fact that Ningxiang pigs have been provided with high-energy diets. In recent years, researchers have been trying to reduce the nutritional level of Ningxiang pigs during the fattening period, probably due to the consideration of carcass traits and the need to conserve feed resources. Our previous study found that a moderate reduction in energy level based on a low protein level ratio could improve slaughter performance and carcass composition of Ningxiang pigs without sacrificing growth performance and IMF content [1], suggesting that frugivorous genes may exist in Ningxiang pigs. This theory assumes that the metabolic thrifty genes in organisms enable the body’s metabolic mechanisms to fully and efficiently utilize limited food and conserve energy as much as possible to cope with food shortage. When food is plentiful, the gene promotes food utilization, fat storage, and rapid weight gain [2].

Indeed, the thrifty gene theory has much to say about pigs as model animals for obesity research [3]. Pigs are very close to humans in anatomical structure, nutritional, and physiological metabolism [4], and are therefore superior to mice in studying the obesity process. Similar pathological reactions occur when consuming high-fat diets, thus making pigs the best animal models for studying fat metabolism associated with high-fat diets [5,6]. More and more studies have proved that pigs, especially the fat-type pigs, are appealing for investigating and understanding the obesity process in humans.

Obesity is a nutritional disorder caused by an imbalance between energy intake and expenditure [7]. It has become a consensus that excessive fat intake leads to obesity, and there is a strong social demand and urgency to study the mechanism of obesity occurrence and then control obesity [8]. Dietary energy levels may have a multidirectional regulatory effect on fat metabolism in pigs, and fat may alter the composition of the intestinal flora in addition to ectopic deposition in muscle and liver [9], which in turn may affect lipid metabolism [10]. Our team’s previous research on Ningxiang pigs found that the intestinal flora can be improved by probiotic intervention, promoting bile acid excretion and thereby reducing liver fat deposition [11]. With the development of metabolomics technology, we can explore the impact of dietary energy changes on lipid metabolism in the body. Therefore, this experiment selected Ningxiang pigs as an animal model to study the changes in carcass composition, digestive function, blood and liver lipid metabolism, gut microbiota, and serum metabolomics of Ningxiang pigs under conditions of excessive or restricted dietary energy in an attempt to reveal its influence mechanism on lipid metabolism. That would help us understand the impact of extreme caloric diets on human health and provide theoretical support for meeting the energy needs of Ningxiang pigs and optimizing carcass composition cost-effectively.

## 2. Materials and Methods

### 2.1. Animal Ethics

All the experimental procedures applied in this study were reviewed and approved by the Animal Care Committee of the Institute, Changsha Medical University, Changsha, China (x2020015). All procedures involving live pigs’ handling, management, and health care followed the regulations for laboratory animals used for scientific purposes.

### 2.2. Experimental Animals and Diets

In an Excel-based animal allocation program (EAAP), a total of 36 healthy Ningxiang pigs with similar age or initial body weight (210 ± 2 d, 43.26 ± 3.21 kg) were randomly allotted into 3 treatments of 6 replicates (1 female and 1 male for each replicate, and the housing size was 2 m × 3 m). Only the test designer was aware of the group allocation at the different stages of the experiment (during the allocation, the conduct of the experiment, the outcome assessment, and the data analysis). According to recommendations of the Chinese National Feeding Standard Type 2 for fatty growing pigs, three trial diets were formulated with similar contents of crude protein (CP) and amino acids (Table 1): control diet (CON, digestive energy (DE): 13.02 MJ/kg), excessive energy diet (EE, DE: 15.22 MJ/kg), and restrictive energy diet (RE, DE: 10.84 MJ/kg). Based on the CON diet, the RE diet used wheat bran as a substitute for rice bran and soybean oil, which were added in a higher quantity in the EE diet, along with peanut meal. During the experiment, aside from dietary variations, the rearing conditions and management protocols were consistent across all treatment groups. The pigs had ad libitum access to feed and water, and routine sanitary measures, such as regular removal of feces, were implemented to maintain hygienic conditions. The trial continued until the pigs in each treatment group reached the optimal slaughter weight (approximately 75 kg) [1]. Pigs in each pen were weighed together at the start and end of each period, and the daily feed consumption per pen was recorded.

### 2.3. Slaughter Survey and Sampling

The week before the trial ended, 8 pigs (4 females and 4 males) were randomly selected from each treatment for jugular vein blood drawing. Immediately following this step, the serum was collected by centrifugation at 4000× *g* for 10 min at 4 °C, treated with liquid nitrogen, and then frozen at −80 °C for metabolomics analysis.

One pig with close-to-average weight was selected from each pen, fasted for 12 h, and then euthanized via electrical stunning followed by exsanguination in accordance with standard commercial protocols. Liver samples were immediately collected; some were stored at −20 °C for chemical analysis, and others were treated with liquid nitrogen and then frozen at −80 °C for RNA extraction. Hot carcass weight was recorded, and the dressing percentage was calculated. Liver was isolated for hepatosomatic index calculation, counted as liver weight divided by carcass weight. Kidney fat percentage, fat percentage, and lean meat percentage were calculated in the same way. Plasma was collected by centrifugation at 4000× *g* for 10 min and stored at −20 °C for further analysis.

### 2.4. Measurement of Parameters Related to Lipid Metabolism in Plasma and Liver

The liver samples were homogenized separately with normal saline and anhydrous ethanol at 1:9 and centrifuged to obtain the supernatant. According to the kit protocol of Nanjing Jiancheng Bioengineering Institute (Nanjing, Jiangsu, China), the contents of low-density lipoprotein cholesterol (LDL-C), triglycerides (TG), total cholesterol (TC), and free fatty acid (NEFA) were determined in anhydrous ethanol homogenate supernatant, and the contents of high-density lipoprotein cholesterol (HDL-C), TP, and total bile acid (TBA) were determined in normal saline homogenate supernatant.

### 2.5. Determination of Apparent Total Tract Digestibility in Nutrients

Air-dried fecal samples and feed samples were determined for dry matter (DM), crude ash (Ash), crude protein (CP), crude fiber (CF), gross energy (GE), and crude fat (EE) content according to the method described in the feed analysis and feed quality testing technology [12].

### 2.6. Analysis of Digestive Enzyme Activity in Intestinal Digesta

Digesta was defrosted at 4 °C and centrifuged at 1000 r/min for 10 min to obtain the supernatant. Lipase (LPS) activity in digesta was measured by a commercial kit (Nanjing Jiancheng Bioengineering Institute, Nanjing, Jiangsu, China). The neutral protease (NP) activity was determined by the Folin phenol method, which is detailed in the Chinese national standard GB/T28715-2012;2012 [13], Standardization Administration of China; Determination of acidic and neutral protease activity in feed additives—Spetrophotometric method.

### 2.7. Quantitative Real-Time PCR Analysis

Total RNA was extracted from each liver sample with TRIZOL reagent (Beijing Solarbio Science Technology Co., Ltd., Beijing, China), checked by Nanodrop (Thermo Scientific, Waltham, MA, USA), and electrophoresed in 1% agarose gel, and reverse-transcribed with RT reagents (Hunan Accurate Biotechnology Co., Ltd., Changsha, Hunan, China) [14]. The PCR cycling condition was as follows: 95 °C for 10 min, 40 cycles at 95 °C for 15 s, 40 cycles at 60 °C for 60 s, and 1 cycle at 72 °C for 30 s. The primers were designed via Primer 5.0 software, as shown in Table 2. The relative expression was expressed as a ratio of the target gene to the control gene according to the 2^−(ΔΔCT)^ method, as reported previously [15].

### 2.8. Analysis of Intestinal Microbial Relative Abundance

Total genomic DNA samples were extracted using E. Z. N. A. Soil DNA Kit (Omega Bio-tek, Inc., Norcross GA, USA) following the manual and stored at −20 °C for subsequent experiments. Concentration and quality of the genomic DNA were checked by NanoDrop 2000 spectrophotometer (Thermo Scientific Inc., Waltham, MA, USA) and agarose gel electrophoresis, respectively. The thermal cycling programming was performed as previously reported [16]. The V3-4 hypervariable region of bacterial 16S rRNA gene was amplified with the universal primer 338F (5′-ACTCCTACGGGAGGCAGCAG-3′) and 806R (5′-GGACTACNNGGGTATCTAAT-3′). The PCR amplicons were purified using an Agencourt AMPure XP Kit (Beckman Coulter, Inc., Brea, CA, USA) and quantified with an ABI StepOnePlus Real-Time PCR System (Applied Biosystems, Inc., Foster City, CA, USA). High-throughput sequencing was performed on an Illumina MiSeq PE300 platform (Illumina, Inc., San Diego, CA, USA) at Beijing Allwegene Technology Co., Ltd. (Beijing, China). After the run, image analysis, base calling, and error estimation were performed using Illumina Analysis Pipeline Version 2.6 (Illumina, Inc., San Diego, CA, USA).

### 2.9. Serum Untargeted Metabolomics Analysis

#### 2.9.1. Metabolites Extraction

In total, 50 uL of serum sample and 200 uL of extract solution (acetonitrile: methanol = 1:1, with isotopically-labeled internal standard mixture) were vortexed for 30 s, sonicated for 10 min in an ice-water bath, then incubated for 1 h at −40 °C, and centrifuged at 12,000 rpm for 15 min at 4 °C to obtain the supernatant. The quality control (QC) sample was prepared by mixing an equal aliquot of the supernatants from all of the samples.

#### 2.9.2. LC-MS/MS Analysis

LC-MS/MS analyses were performed using a UHPLC system (Vanquish, Thermo Fisher Scientific, Waltham, MA, USA) with a UPLC BEH Amide column (2.1 mm × 100 mm, 1.7 μm). The mobile phase consisted of 25 mmol/L ammonium acetate and 25 ammonia hydroxide in water (pH = 9.75) (A) and acetonitrile (B). According to the method reported and validated previously [17,18]. The analysis was carried out with an elution gradient as follows: 0~0.5 min, 95%B; 0.5~7.0 min, 95%~65% B; 7.0~8.0 min, 65%~40% B; 8.0~9.0 min, 40% B; 9.0~9.1 min, 40%~95% B; 9.1~12.0 min, 95% B. The column temperature was 25 °C. The auto-sampler temperature was 4 °C, and the injection volume was 3 uL.

MS/MS spectra were acquired on information-dependent acquisition (IDA) mode in the control of the acquisition software (Xcalibur4.1, Thermo Fisher Scientific, Waltham, MA, USA) using a QE HFX mass spectrometer (Orbitrap MS, Thermo). The ESI source conditions were set as follows: sheath gas flow rate as 50 Arb, aux gas flow rate as 10 Arb, capillary temperature 320 °C, full MS resolution as 60,000, MS/MS resolution as 7500, collision energy as 10/30/60 in NCE mode, spray voltage as 3.5 kV (positive) or −3.2 kV (negative), respectively.

#### 2.9.3. Data Preprocessing and Annotation

The raw data were converted to the mzXML format using ProteoWizard v3.0.20287(64-bit) and processed with an in-house R program based on XCMS [19]. Then, an in-house MS2 database (BiotreeDB) was applied in metabolite annotation. The cutoff for annotation was set at 0.3.

### 2.10. Statistical Analyses

The one-way analysis of variance (ANOVA) with Levene’s test was used for all comparisons except serum metabolomics comparisons between two groups analyzed by two-tailed unpaired Student’s *t* tests (parametric) (IBM SPSS 23 software, Chicago, IL, USA). The level of statistical significance was set at *p* < 0.05.

## 3. Results

### 3.1. Carcass Composition

As shown in Table 3, the fat percentage and hepatosomatic index of the EE group were significantly increased compared with the other two groups (*p* < 0.05). There was no difference in other slaughter performance parameters (*p* > 0.05).

### 3.2. Apparent Total Tract Digestibility and Digestive Enzyme Activity

Effects of excessive or restrictive energy on apparent total tract digestibility (ATTD) and digestive enzyme activity were shown in Table 4. The apparent digestibility of CP and GE in group EE was significantly higher than that in the other two groups (*p* < 0.01). The apparent digestibility of Ash, DM, and EE was significantly increased with the increase in energy level (*p* < 0.01). The activities of NP and LPS in the jejunum of the EE group were significantly higher than those of the other two groups (*p* < 0.05), and the LPS activity in the jejunum of the EE group was significantly higher than that of the CON group (*p* < 0.05).

### 3.3. Parameters Related to Lipid Metabolism in Plasma and Liver

Table 5 showed no difference in lipid metabolism indexes in serum (*p* > 0.05). The content of NEFA, TG, and TC in the liver of the RE group was significantly decreased compared with the other two groups (*p* < 0.01). The content of LDL-C in the liver of the EE group was observed to be higher than in the other two groups (*p* < 0.01), and the TBA content tended to be raised.

### 3.4. Expression of Gene Related to Lipid Metabolism in Liver

**Figure 1:**
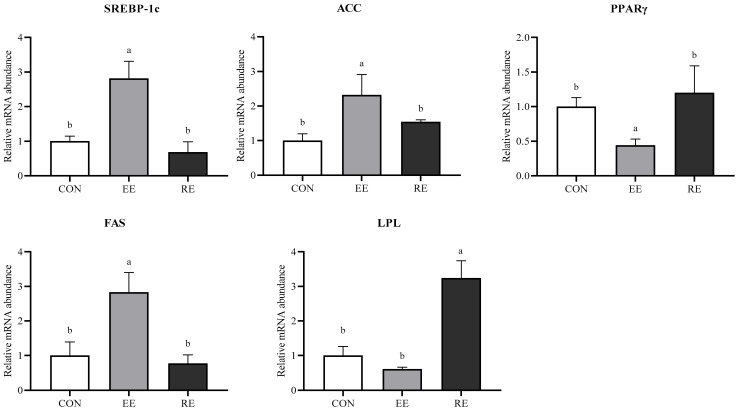
Effects of excessive or restrictive energy on gene expression levels in the liver of Ningxiang pigs. Data were expressed as means ± SEM (*n* = 6). CON—control diet group. EE—excessive energy diet group. RE—restrictive energy diet group. Values with different letters are significantly different among the three groups (*p* < 0.05). Sterol regulatory element-binding protein-1c (*SREBP-1c*), acetyl CoA carboxylase (*ACC*), peroxisome proliferator-activated receptor γ (*PPARγ*), fatty acid synthase (FAS), and lipoprotein lipase (*LPL*).

As shown in Figure 1, the mRNA expression of acetyl-CoA carboxylase (*ACC*), fatty acid synthase (*FAS*), and sterol regulatory element-binding protein-1C (*SREBP-1C*) in the liver was up-regulated in the EE group (*p* < 0.05), while the mRNA expression of lipoprotein lipase (*LPL*) in liver was up-regulated in the RE group (*p* < 0.05).

### 3.5. Intestinal Microbial Relative Abundance

Significant variation in phylum-level and genus-level abundance (>1%) in gut microbiota of pigs fed excessive or restrictive energy diets was shown in Table 6. Compared with the control group, the abundance of *p_Bacteroidetes* in experimental groups was significantly decreased (*p* < 0.05), while the abundance of *p_Firmicutes* was significantly increased (*p* < 0.05), and the ratio of *Firmicutes* to *Bacteroidetes* was significantly increased in the RE group (*p* < 0.05). Compared with the other two groups, the abundance of *g_Terrisporobacter* in the EE group was significantly decreased (*p* < 0.05), and the abundance of *g_Clostridium_sensu_stricto_1* was significantly increased (*p* < 0.05). The abundance of *g_Streptococcus* in the RE group was significantly increased (*p* < 0.05), compared with that in the other two groups (*p* < 0.05).

### 3.6. Serum Untargeted Metabolomics

**Figure 2:**
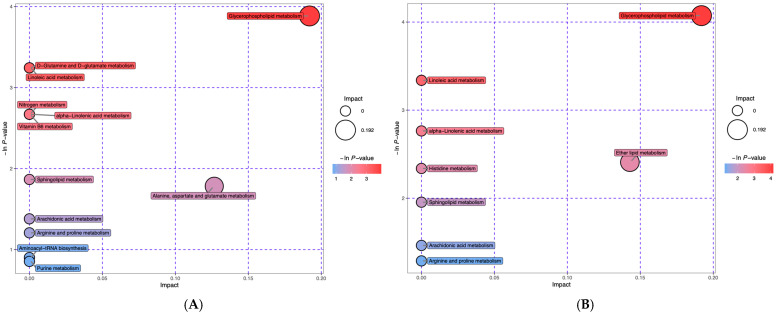
Pathway analysis for group EE vs. CON (**A**) and RE vs. CON (**B**) expressed as bubble plots. Significantly changed pathways based on the enrichment and topology analysis are shown. The *x*-axis represents pathway enrichment, and the *y*-axis represents the pathway impact. Large sizes and dark colors represent the major pathway enrichment and high pathway impact values, respectively. CON—control diet group. EE—excessive energy diet group. RE—restrictive energy diet group.

**Figure 3:**
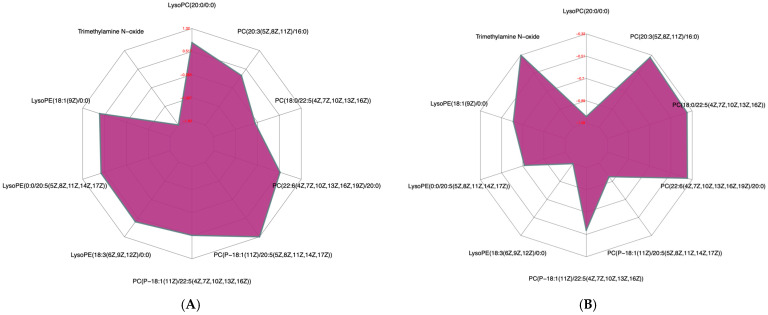
Radar chart analysis comparing the EE and CON groups (**A**), and the RE and CON groups (**B**). For each comparison, the corresponding ratio of the quantitative values of the differential metabolites was calculated and subjected to a logarithmic transformation with base 2. The corresponding content trend change is displayed in a radar chart. CON—control diet group; EE—excessive energy diet group; RE—restrictive energy diet group.

The 45 differential metabolites in groups EE vs. RE and the 54 differential metabolites in groups RE vs. CON were obtained (results omitted). Metabolomics pathway analysis (MetPA) was conducted through commercial databases, including KEGG and MetaboAnalyst, and visualized via an interactive visualization framework (Figure 2). The metabolic pathways with impact value > 0.1 or −log (p) > 10 were considered the most relevant pathways involved in the conditions under study [20]. Hence, this study was mainly focused on glycerophospholipid metabolism. Table 7 summarizes nine identical serum differential metabolites in groups EE vs. CON and groups RE vs. CON, which belonged to glycerophospholipids, and a radar chart was drawn based on fold change values, respectively (Figure 3). Relative to the CON group, eight of them were up-regulated in the EE group but down-regulated in the RE group, one of them (LysoPC (20:0/0:0), KEGG ID: C04230) hit the pathway of glycerophospholipid metabolism. Furthermore, one of phosphatidylcholine (PC (18:0/22:5(4Z,7Z,10Z,13Z,16Z)), KEGG ID: C00157) was found to be significantly down-regulated in experimental groups relative to the CON group, while another phosphatidylcholine (PC (20:3(5Z,8Z,11Z)/16:0), KEGG ID: C00157) was up-regulated in groups EE vs. CON and down-regulated in groups RE vs. CON. These phosphatidylcholines were also connected to the pathway of glycerophospholipid metabolism. In addition, trimethylamine N-oxide (TMAO) was significantly down-regulated in groups EE vs. CON and unchanged in groups RE vs. CON.

## 4. Discussion

### 4.1. EE-Induction Strengthens Lipogenic Gene Expression and Bile Acid Metabolism May Account for Increased Body Fat Deposition and Hepatic Lipid Accumulation in Ningxiang Pigs

Studies have reported that high-fat diets increased serum lipid levels (including free fatty acids (FFA or NEFA), TG, TC, phospholipids, and lipoproteins) in piglets, whereas restricted diets decreased TG, LDL-C, and phospholipids [21]. In this study, serum lipid indexes did not change significantly with different energy levels. However, the liver index of the EE group was significantly increased, and TG and NEFA levels in the liver were also elevated, but neither was significant. When the supply of fuel molecule NEFA in the blood is sufficient, it is partially consumed by hepatic uptake and metabolism; when the supply exceeds the oxidative capacity of the liver, the excess NEFA is esterified to TG, reassembled into very low-density lipoprotein cholesterol (VLDL), secreted into the bloodstream for transport to extrahepatic tissues, or stored in the liver. Therefore, the changes in NEFA in the liver reflect the lipid metabolism of the liver to a certain extent. SREBP-1c activates lipogenic genes *ACC* and *FAS* [22] and plays an important role in regulating intracellular cholesterol and fatty acids [23]. In this study, mRNA expressions of *ACC*, *FAS*, and *SREBP-1c* were significantly up-regulated in the EE group, indicating that EE increased de novo synthesis of fatty acids in the liver. TG and NEFA levels were significantly reduced, and *LPL* mRNA expression was significantly up-regulated in the RE group, suggesting that RE promoted fatty acid β-oxidation and ketone body formation. Also, Sejersen et al. [20] reported that the hepatic response of growing pigs to restricted diets exhibited increased gluconeogenesis and glycogenolysis. LPL hydrolyzes the triglycerides carried by lipoproteins and releases NEFA. When dietary energy restriction resulted in insufficient hepatic intake of NEFA from the bloodstream, the expression of *LPL* mRNA in the liver was up-regulated to release more endogenous NEFA for gluconeogenesis, which increases the NEFA consumption and reduces the TG deposition in the liver. Ji et al. also proposed that lipolysis occurs preferentially by modulating AMP-activated protein kinase (AMPK) activity during transient nutrient deprivation [24].

LDL-C content and TBA content were significantly increased in the EE group with no change in TC content, which shows that excessive dietary energy may increase endogenous cholesterol synthesis in the liver and TBA content, as cholesterol is known to be a precursor for bile acid synthesis. Conversely, the RE group had significantly lower TC levels, no change in TBA levels, and significantly higher HDL-C levels. Blood TC level has been reported to be associated with hypertrophymia [25]. HDL-C is produced in the liver and is responsible for returning cholesterol from various tissues to the liver for metabolism [26]. Accordingly, it is speculated that a restrictive energy diet may reduce endogenous cholesterol synthesis in the liver and promote cholesterol reverse transport for bile acid synthesis, while excessive energy increases endogenous cholesterol synthesis and bile acid synthesis, and secretion. As an important emulsifier of lipid molecules [27], bile acids activate lipase zymogen and increase the lipase activity. The results of increased lipase activity in the jejunum and ileum of the EE group in this study also proved that excessive energy increased bile acid synthesis, activating more lipase zymogen into lipase in the small intestine [28], which also explains the increase in fat digestibility in the present study.

### 4.2. Dietary Energies Improved Host Energy Utilization by Increasing the Ratio of Firmicutes to Bacteroides, and EE-Induced Increase in the Abundance of g_Clostridium May Be Related to Liver Bile Acid Metabolism

High-fat diets are known to enhance biliary secretion and promote lipid digestion, and bile acid dysregulation is associated with obesity [29]. When dietary energy is excessive, bile acids synthesized by the liver alone are far from sufficient to emulsify the large amount of fat ingested. And the “enterohepatic circulation” of bile acids can compensate for this deficiency. For one thing, bile acids inhibit the proliferation of harmful bacteria and regulate the composition of intestinal flora [30]. For another, the metabolism of intestinal flora affects the expression of enzymes related to bile acid synthesis [31]. Bile salt hydrolases are enzymes specific to intestinal flora that affect bile acid metabolism by unfolding bound bile acids into free bile acids [32]. In this study, the relative abundance of *Terrisporobacter* was decreased in the colon of the EE group, which may be related to the bacteriostasis effect of bile acids. And yet the bacteriostatic effect of bile acids is selective, as it inhibits the growth of bile acid-intolerant bacteria but promotes the proliferation of bacteria tolerant to high concentrations of bile acids [33], for instance, leading to the overgrowth of several bacteria in *Clostridia* [34]. *Clostridia* have been found to contribute to the 7α dehydroxylation of primary bile acids [35,36]. For example, *Clostridium scindens* directly converts primary bile acids into secondary bile acids [37], thereby modulating *Farnesoid X receptor* (FXR)/Takeda G-protein-coupled receptor 5 (TGR5) signaling that governs hepatic lipogenesis and glucose handling [38]. This provides a mechanistic route by which bile acid–tolerant *Clostridia* can influence host hepatic metabolism [39,40]. In humans, multi-omics studies have linked gut microbial functions and metabolites to hepatic steatosis and even showed that a microbial metabolite (phenylacetic acid) can drive steatosis features, supporting a causal microbiota–liver axis. The increased abundance of the dominant bacterium *Clostridium_sensu_stricto_1* in the EE group may be due to overproduction of hepatic bile acids; conversely, it was the proliferation of *Clostridium_sensu_stricto_1* that may boost the metabolism of bile acids in the host.

An increased ratio of *Firmicutes* to *Bacteroides* in the gut has been reported in obese animals [41] and has been associated with host energy intake [42,43]. This study showed that the restrictive energy diet significantly increased the ratio of *Firmicutes* to *Bacteroidetes*, whereas Turnbaugh et al. [44] previously pointed out that the ratio is positively correlated with body fat percentage. It has also been reported that during the restricted feeding period in pigs, most energy intake was stored in the form of protein rather than fat [45] and the reduction in adipose tissue deposition is more pronounced; after compensatory feeding, it is adipose tissue that exhibits compensatory growth rather than muscle tissue in pigs [46,47]. A new explanation may be derived from the findings in the present study: a restrictive energy diet increased the abundance of *Firmicutes* and the ratio *Firmicutes* to *Bacteroides*, which improved the energy utilization of the host. Thus, growth was not significantly restricted, and adipose tissue deposition was decreased, but susceptibility to obesity was increased; restricted Ningxiang pigs were liable to deposit fat by the action of intestinal flora once they were compensated for energy.

### 4.3. Dietary Energy Changes Affected Hepatic Lipid Metabolism by Modulating the Glycerophospholipid Metabolism Pathway, and EE Also May Be Involved in This Pathway by Altering the Gut Microbial Metabolite TMAO

The carbon chain types of lipid biomarkers obtained in this experiment mainly included C18, C20, and C22, which are the main sources of arachidonic acid (C20:4), an important inflammatory mediator [48]. Changes in these phosphatidylcholine (PC) and phosphatidylethanolamine (PE) substances in serum indicated that excessive or restrictive energy diet may cause the metabolic disorder of inflammatory mediators. As the main phospholipid component in all plasma lipoproteins [49,50] PC is physiologically important in lipoprotein metabolism. The liver is a major site for PC synthesis and plasma lipoprotein production, serving as both a donor of PCs during lipoprotein assembly and secretion and an acceptor of PCs in plasma lipoproteins [51], and its PC biosynthesis regulates the amounts of circulating lipoproteins. In metabolic dysfunction-associated steatotic liver disease (MASLD), an oversupply of fatty acids—both from diet and de novo synthesis—overloads mitochondria, crippling β-oxidation and jamming VLDL assembly and export [52]. Impaired PC biosynthesis in the liver greatly reduces VLDL and HDL levels in circulation and reduces plasma HDL levels by inhibiting HDL formation in the liver and increasing hepatic uptake of HDL from the circulation [50]. Conversely, compensatory enhancement of PC synthesis may promote hepatic production of HDL and supply of HDL to the blood, thus facilitating the reverse transport of cholesterol in the blood into the liver. In this study, an excessive energy diet increased the relative amount of PC, which may explain the increase in hepatic TBA and may also be a result of compensatory enhancement of hepatic cholesterol metabolism. Meanwhile, no significant changes in blood lipids were observed, which may be related to the absorption and deposition of lipids in the liver. According to Vance et al. [51], half of the PC delivered from lipoproteins to the liver was converted into triacylglycerol. In contrast to the elevation of PC caused by an excessive energy diet, a restrictive energy diet decreased the relative amount of PC, suggesting that excessive or restrictive energy diets may affect hepatic lipid metabolism by modulating the glycerophospholipid metabolic pathway, with a potential biomarker of PC (20:3(5Z,8Z,11Z)/16:0) (KEGG ID: C00157).

Intestinal flora has been shown to influence lipid biosynthesis [41] and lipid degradation [53] in the liver of the host, particularly affecting the distribution of certain TG and PC [54]. Notably, TMAO in this study was down-regulated in the EE group and unchanged in the RE group. He et al. [55] found that the serum TMAO concentration of Ningxiang pigs was significantly lower than that of lean-type pigs. TMAO has been previously reported to be associated with the function of the gut microbiota [56,57], especially the regulation of host lipid metabolism by the gut microbiota [58]. PC can be converted into choline and trimethylamine in the presence of gut microbiota, the latter of which passes through the portal vein into the liver and is metabolized to TMAO [59]. Caesar et al. [60] have pointed out that the regulation of hepatic cholesterol metabolism by gut microbiota depends on dietary lipid composition and that choline-rich diets upregulate tumor TMAO levels by commensal microbiota [50]. In this study, excessive energy up-regulated the relative content of PC in serum and down-regulated the relative content of TMAO, which may be connected with the reduction in certain bacteria producing trimethylamine lyase in the intestine, indicating that the changes in dietary energy may affect the PC metabolism through modulation of the gut microbiota. In line with this notion, human evidence indicates that circulating TMAO is associated with altered cholesterol handling, atherosclerosis risk, and steatosis progression, implying that microbiota-dependent TMAO can influence hepatic lipid metabolism in vivo [61,62,63].

Pigs (including Ningxiang pigs) share gastrointestinal anatomy, lipid digestion, and lipoprotein profiles broadly comparable to humans; nevertheless, breed-specific energetics and dietary lipid composition differ and should be acknowledged when extrapolating to human nutrition [61,64]. Even so, similar microbiota–lipid interactions have been reported in human cohorts with steatosis/obesity [62,65], suggesting that managing dietary energy alongside bile acid–microbiota pathways may be relevant for human dietary guidance.

## 5. Conclusions

The findings suggest that a restrictive energy diet increased the abundance of Firmicutes and the ratio Firmicutes to Bacteroides, which improved the energy utilization of the host, causing no obvious negative effect on carcass traits of Ningxiang pigs, although it may increase susceptibility to obesity. Excessive energy increased the digestibility of nutrients and digestive enzyme activity, possibly mediated through the perturbation of g_Clostridium, shaping the bile acid metabolism, thereby exacerbating body fat deposition in Ningxiang pigs. Excessive energy also up-regulated the expression of lipogenic genes, resulting in fat accumulation in the liver. Dietary energy changes may affect hepatic lipid metabolism by regulating the glycerophospholipid metabolic pathway and phosphatidylcholines such as PC (20:3(5Z,8Z,11Z)/16:0) (KEGG ID: C00157) may be potential biomarkers. The alterations in intestinal microbial metabolite TMAO caused by excessive energy may also represent a pathway through which it is involved in glycerophospholipid metabolism.

### Limitations of the Study

Indeed, it cannot be ignored that the relatively small sample size may lead to potential limitations in extrapolating the findings in the present study. Furthermore, to obtain the same protein content and amino acids balance in diets, especially when formulating diets at extreme energy levels, it is inevitable that there will be slight differences in protein sources. Thus, the use of different protein sources may produce other effects. While pigs are valuable translational models, differences in dietary lipid composition and energy metabolism between Ningxiang pigs and humans should be acknowledged. Therefore, caution is warranted when extrapolating the microbial–lipid interactions observed in this study to humans.

## Figures and Tables

**Table 1:** Ingredient and chemical composition of experimental diets (air-dry matter basis) ^1^.

Ingredient, %	CON	EE	RE
Corn	54.90	57.10	47.90
Soybean meal	8.20	5.60	8.20
Peanut meal	0.00	5.00	0.00
Wheat bran	21.30	0.00	29.8
Rice bran	6.40	21.40	0.00
Soybean oil	3.20	7.90	0.00
CaHPO4	0.27	0.51	0.19
Limestone	1.01	0.90	1.05
L-Lys HCl 98%	0.11	0.11	0.13
Threonine	0.01	0.00	0.03
Tryptophan	0.00	0.02	0.00
Rice chaff	1.7	0.46	1.5
Zeolite	1.90	0.00	10.20
Premix ^2^	1.00	1.00	1.00
Total	100.00	100.00	100.00
Nutrient levels ^3^			
Digestible energy, MJ/kg	13.02	15.22	10.84
Crude protein	12.01	12.01	12.01
Crude fiber	4.07	3.31	4.06
Crude fat	5.28	9.40	2.12
Lys	0.60	0.60	0.60
Met	0.20	0.20	0.20
Thr	0.44	0.44	0.44
Try	0.13	0.12	0.13
Calcium	0.50	0.50	0.50
Total phosphorus	0.53	0.61	0.49
Available phosphorus	0.16	0.16	0.16

^1^ Basal diet formulated according to the Chinese National Feeding Standard for Swine; ^2^ supplied, per kilogram of diet: 0.05 mg Cu; 0.03 mg I; 0.8 mg Fe; 0.002 mg Se; 0.8 mg Zn; 0.06 mg Mn; 1.3 mg vitamin K (menadione); 2 mg vitamin B1; 5.8 mg vitamin B2; 18.79 mg vitamin B3; 14.5 μg vitamin B12; 3324 IU vitamin A; 376 IU vitamin D; 28.9 IU vitamin E; 80 mg choline chloride; 200 mg antioxidants; 500 mg fungicide; ^3^ The contents of crude protein and crude fiber were analyzed. CON—control diet group. EE—excessive energy diet group. RE—restrictive energy diet group.

**Table 2:** Primers used for quantitative real-time PCR.

Gene	Accession No.	Primer Sequence (5′-3′)	Product Size (bp)
*SREBP-1c*	NM_214157.1	F: GCGACGGTGCCTCTGGTAGT	96
		R: CGCAAGACGGCGGATTTA	
*LPL*	NM 214286	F: ATCTGCGGGATACACCAAGC R: CCAAGGCTGTATCCCAGGAG	110
*ACC*	NM-001114269	F: GGCCATCAAGGACTTCAACC	120
		R: ACGATGTAAGCGCCGAACTT	
*FAS*	NM-001099930	F: ACACCTTCGTGCTGGCCTAC	112
		R: ATGTCGGTGAACTGCTGCAC	
*PPAR γ*	NM-214379	F: GAGGGCGATCTTGACAGGAA	124
		R: GCCACCTCTTTGCTCTGCTC	
*β-actin*	XM-003124280.3	F: CCTGCGGCATCCACGAAAC	123
		R: TGTCGGCGATGCCTGGGTA	
*GAPDH*	NM-001206359.1	F: TCGGAGTGAACGGATTTGGC	95
		R: GAAGGGGTCATTGATGGCGA	

*SREBP-1c*—sterol regulatory element-binding protein-1c; *LPL*—lipoprotein lipase; *ACC*—acetyl-CoA carboxylase; *FAS*—fatty acid synthase; *PPARγ*—peroxisome proliferator-activated receptor gamma; *β-actin*—beta-actin; *GAPDH*—glyceraldehyde-3-phosphate dehydrogenase.

**Table 3:** Effects of excessive or restrictive energy on carcass composition and hepatosomatic index of Ningxiang pigs.

Parameters	CON	EE	RE	SEM	*p*-Value
Dressing percentage/%	73.95	73.43	72.34	0.71	0.678
Kidney fat percentage/%	5.06	6.04	5.57	0.38	0.603
Fat percentage/%	39.50 ^b^	42.98 ^a^	38.77 ^b^	0.74	0.047
Lean meat percentage/%	39.53	38.20	39.38	0.60	0.663
Backfat thickness/mm	37.42	42.93	36.53	1.66	0.290
Hepatosomatic index/%	1.65 ^b^	2.17 ^a^	1.68 ^b^	0.09	0.018

Data are presented as means ± SEM (*n* = 6). ^a,b^ Mean values within a row with different superscript letters indicate significant differences (*p* < 0.05). CON—control diet group. EE—excessive energy diet group. RE—restrictive energy diet group.

**Table 4:** Effects of excessive or restrictive energy on apparent total tract digestibility (ATTD) and digestive enzyme activity of Ningxiang pigs.

Parameters	CON	EE	RE	SEM	*p*-Value
apparent total tract digestibility (ATTD)/%					
CP	71.60 ^b^	80.94 ^a^	72.22 ^b^	1.55	0.005
Ash	72.50 ^b^	81.91 ^a^	68.31 ^c^	1.82	0.000
DM	70.76 ^b^	80.41 ^a^	66.07 ^c^	1.92	0.000
GE	75.79 ^b^	83.75 ^a^	75.40 ^b^	1.3	0.001
EE	55.79 ^b^	71.21 ^a^	40.72 ^c^	3.94	0.000
Jejunum					
LPS/(U/dl)	74.59 ^b^	200.39 ^a^	107.25 ^a,b^	23.06	0.043
NP/(U/mL)	123.47 ^b^	371.21 ^a^	137.48 ^b^	46.93	0.049
Ileum					
LPS/(U/dl)	46.43 ^b^	79.86 ^a^	31.58 ^b^	7.89	0.016
NP/(U/mL)	181.95	247.11	174.69	28.95	0.591

Data are presented as means ± SEM (*n* = 6). ^a,b,c^ Mean values within a row with different superscript letters indicate significant differences (*p* < 0.05). CON—control diet group. EE—excessive energy diet group. RE—restrictive energy diet group.

**Table 5:** Effects of excessive or restrictive energy on lipid metabolism in the serum and liver of Ningxiang pigs.

Parameters	CON	EE	RE	SEM	*p*-Value
Serum					
TG	0.57	0.88	0.67	0.88	0.396
TC	3.67	4.13	3.19	0.2	0.169
HDL-C (mmol/L)	1.49	1.53	1.13	0.09	0.120
LDL-C (mmol/L)	1.65	1.72	1.62	0.1	0.922
HDL-C/LDL-C	0.94	0.9	0.76	0.06	0.415
Liver					
HDL-C (mmol/gprot)	14.21 ^b^	20.47 ^b^	31.38 ^a^	2.41	0.002
TBA (umol/gprot)	3.91 ^b^	7.07 ^a^	5.98 ^a,b^	0.56	0.041
NEFA (mmol/L)	0.24 ^a^	0.34 ^a^	0.10 ^b^	0.03	0.006
TG (mmol/L)	1.26 ^a^	1.60 ^a^	0.88 ^b^	0.10	0.006
LDL-C(mmol/L)	0.43 ^b^	0.65 ^a^	0.34 ^b^	0.04	0.000
TC (mmol/L)	0.89 ^a^	0.87 ^a^	0.71 ^b^	0.03	0.001

Data are presented as means ± SEM (*n* = 6). ^a,b^ Mean values within a row with different superscript letters indicate significant differences (*p* < 0.05). CON—control diet group. EE—excessive energy diet group. RE—restrictive energy diet group.

**Table 6:** Effects of excessive or restrictive energy on the abundance of gut microbiota in Ningxiang pigs, % (*n* = 4).

Parameters	CON	EE	RE	SEM	*p*-Value
phylum level					
*p__Firmicutes*	74.97 ^b^	85.44 ^a^	88.42 ^a^	1.97	0.001
*p__Bacteroidetes*	8.11 ^a^	3.81 ^b^	2.78 ^b^	0.89	0.019
*p__Firmicutes/Bacteroidetes*	10.06 ^b^	24.53 ^a,b^	34.00 ^a^	3.85	0.024
genus level					
*p__Firmicutes; c__Bacilli; o__Lactobacillales; f__Streptococcaceae*					
*g* *__Streptococcus*	2.82 ^b^	1.59 ^b^	11.65 ^a^	1.75	0.012
*p__Firmicutes; c__Clostridia; o__Clostridiales; f__Clostridiaceae_1*					
*g* *__Clostridium_sensu_stricto_1*	11.30 ^b^	19.48 ^a^	11.50 ^b^	1.59	0.017
*p__Firmicutes; g__ c__Clostridia;o__Clostridiales; f__Peptostreptococcaceae*					
*g* *__Terrisporobacter*	8.78 ^a^	3.86 ^b^	9.74 ^a^	1.07	0.035
*p__Firmicutes; c__Clostridia; o__Clostridiales; f__Lachnospiraceae*					
*g* *__Lachnospiraceae_XPB1014_group*	3.08	6.05	1.50	0.83	0.050

Data are presented as means ± SEM (*n* = 4). ^a,b^ Mean values within a row with different superscript letters indicate significant differences (*p* < 0.05). CON—control diet group. EE—excessive energy diet group. RE—restrictive energy diet group.

**Table 7:** Serum differential metabolites in group EE vs. CON and group RE vs. CON (*n* = 8).

Metabolites	Biological Role	EE	RE
LysoPE (0:0/20:5(5Z,8Z,11Z,14Z,17Z))	Glycerophospholipids	**+**	**-**
LysoPE (18:1(9Z)/0:0)	Glycerophospholipids	**+**	**-**
LysoPE (18:3(6Z,9Z,12Z)/0:0)	Glycerophospholipids	**+**	**-**
PC (P-18:1(11Z)/22:5(4Z,7Z,10Z,13Z,16Z))	Glycerophospholipids	**+**	**-**
PC (P-18:1(11Z)/20:5(5Z,8Z,11Z,14Z,17Z))	Glycerophospholipids	**+**	**-**
PC (22:6(4Z,7Z,10Z,13Z,16Z,19Z)/20:0)	Glycerophospholipids	**+**	**-**
LysoPC (20:0/0:0) (C04230)	Glycerophospholipids	**+**	**-**
PC (18:0/22:5(4Z,7Z,10Z,13Z,16Z)) (C00157)	Glycerophospholipids	**-**	**-**
PC (20:3(5Z,8Z,11Z)/16:0) (C00157)	Glycerophospholipids	**+**	**-**

CON—control diet group. EE—excessive energy diet group. RE—restrictive energy diet group. **+**, **-** indicate up-regulation and down-regulation relative to the CON group, respectively.

## Data Availability

The data presented in this study are available on request from the corresponding authors.
